# Supplementary figures and images for: Synaptic and peptidergic connectome of a neurosecretory center in the annelid brain
Source: eLife. 2017 Dec 4;6:e26349. doi: 10.7554/eLife.26349 (PMC5747525; doi:10.7554/eLife.26349)

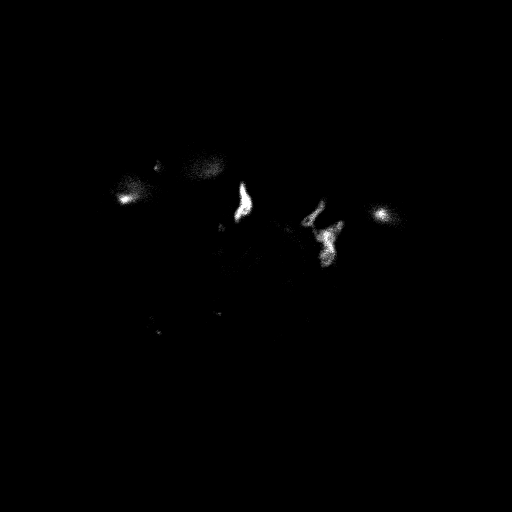

Supplement: Figure 3—figure supplement 5—source data 1. [file elife-26349-fig3-figsupp5-data1.zip › 3-day-old_acTub_anterior.tif]
