# Supplementary material for: Synaptic and peptidergic connectome of a neurosecretory center in the annelid brain
Source: eLife. 2017 Dec 4;6:e26349. doi: 10.7554/eLife.26349 (PMC5747525; doi:10.7554/eLife.26349)
Supplement: Supplementary file 36 [file elife-26349-fig1-data1.odt]

Figure 1-source data 1
